# PGA: a software package for rapid, accurate, and flexible batch annotation of plastomes

**DOI:** 10.1186/s13007-019-0435-7

**Published:** 2019-05-21

**Authors:** Xiao-Jian Qu, Michael J. Moore, De-Zhu Li, Ting-Shuang Yi

**Affiliations:** 1 Germplasm Bank of Wild Species, Kunming Institute of Botany, Chinese Academy of Sciences, 132 Lanhei Road, Kunming, 650204 Yunnan China; 2 Shandong Provincial Key Laboratory of Animal Resistance Biology, Institute of Biomedical Sciences, College of Life Sciences, Shandong Normal University, Jinan, Shandong China; 3 Department of Biology, Oberlin College, Oberlin, OH USA

**Keywords:** PGA, Plastome, Batch annotation, Accuracy, BLAST, Software, Algorithms

## Abstract

**Background:**

Plastome (plastid genome) sequences provide valuable information for understanding the phylogenetic relationships and evolutionary history of plants. Although the rapid development of high-throughput sequencing technology has led to an explosion of plastome sequences, annotation remains a significant bottleneck for plastomes. User-friendly batch annotation of multiple plastomes is an urgent need.

**Results:**

We introduce Plastid Genome Annotator (PGA), a standalone command line tool that can perform rapid, accurate, and flexible batch annotation of newly generated target plastomes based on well-annotated reference plastomes. In contrast to current existing tools, PGA uses reference plastomes as the query and unannotated target plastomes as the subject to locate genes, which we refer to as the reverse query-subject BLAST search approach. PGA accurately identifies gene and intron boundaries as well as intron loss. The program outputs GenBank-formatted files as well as a log file to assist users in verifying annotations. Comparisons against other available plastome annotation tools demonstrated the high annotation accuracy of PGA, with little or no post-annotation verification necessary. Likewise, we demonstrated the flexibility of reference plastomes within PGA by annotating the plastome of *Rosa roxburghii* using that of *Amborella trichopoda* as a reference. The program, user manual and example data sets are freely available at https://github.com/quxiaojian/PGA.

**Conclusions:**

PGA facilitates rapid, accurate, and flexible batch annotation of plastomes across plants. For projects in which multiple plastomes are generated, the time savings for high-quality plastome annotation are especially significant.

## Background

The plastid genomes (plastomes) of most photosynthetic seed plants are highly conserved and have a quadripartite structure with a large and a small single-copy regions separated by two inverted repeat (IR) regions [1, 2]. The plastomes of photosynthetic seed plants are usually 120–160 kb [1] in size and contain 101–118 unique genes [2]. Plastome sequences have been widely applied in phylogenetics [3–5], population genetics and phylogeography [6, 7], and comparative genomics [2, 8]. In addition, the plastome is a key target for genetic engineering efforts to improve economic traits, resistance to diseases and pests, and stress resistance [9, 10].

The rapid development of high-throughput sequencing platforms has led to an explosion of plastome sequence data, especially via genome skimming approaches [11]. However, annotation of plastomes remains a significant bottleneck, especially if users wish to batch annotate multiple plastomes. Existing tools for plastome annotation include four web servers (DOGMA [12], CpGAVAS [13], Verdant [14] and GeSeq [15]) and one command line tool (Plann [16]). However, gene annotations from these programs should be checked manually, and potentially inaccurate gene annotations are not always flagged for checking. Hence batch annotation of plastomes using these tools may still be a time-consuming task.

**Fig. 1:**
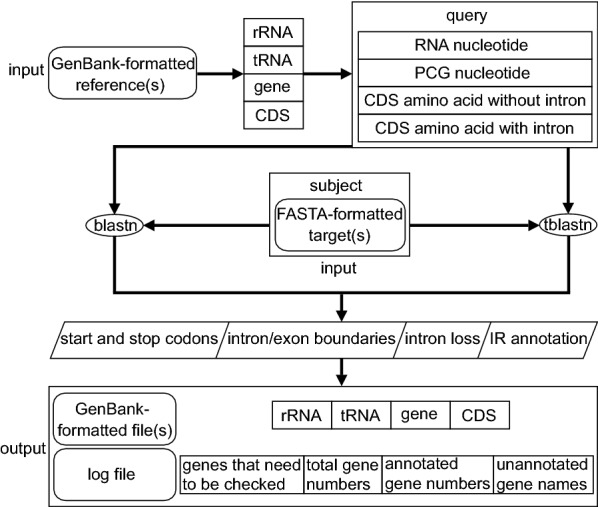
PGA annotation flowchart. See text for detailed information on each step

**Fig. 2:**
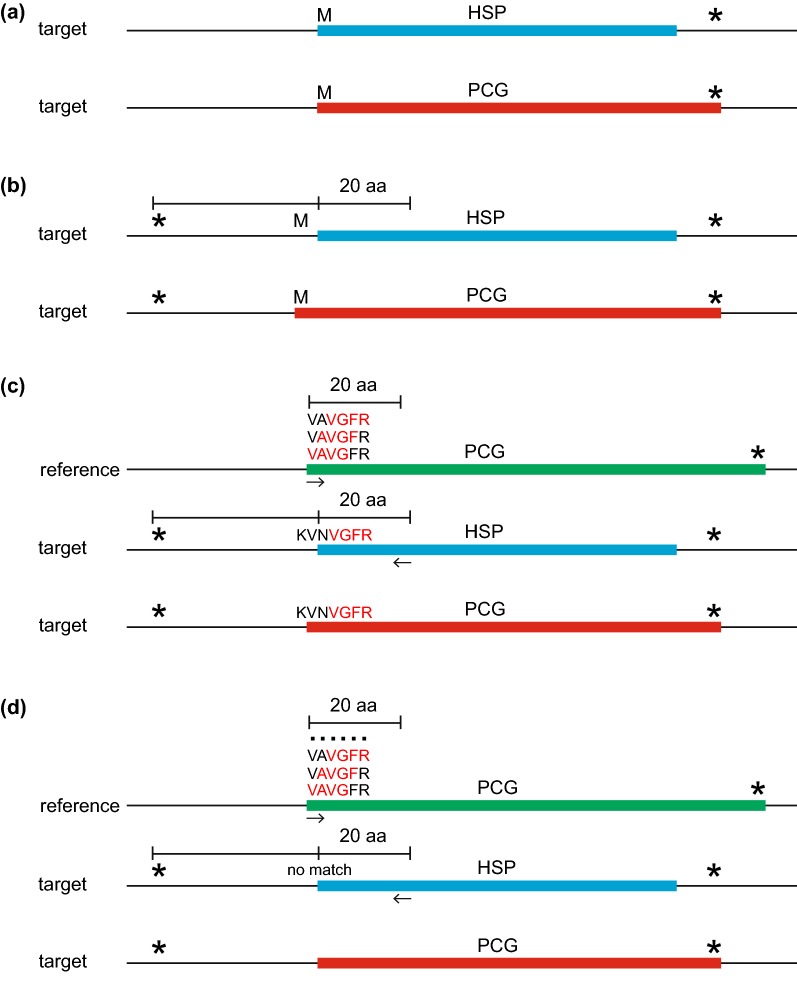
A description of the Gene Boundary Detection Algorithm (GBDA), which determines start and stop codons for Protein-Coding Genes (PCGs). Panels (**a**) through (**d**) correspond to the description of this algorithm in the “Boundary detection algorithm” section of the main text. The blue bar denotes the original HSP resulting from TBLASTN search of the reference CDS. The red bar denotes the annotated PCG resulting from this algorithm. The green bar denotes the reference CDS with the highest percent identity to the target plastome. The “M” (methionine) and “*” denote the start (ATG) and stop (TAA/TAG/TGA) codons in the same reading frame as the HSP

**Fig. 3:**
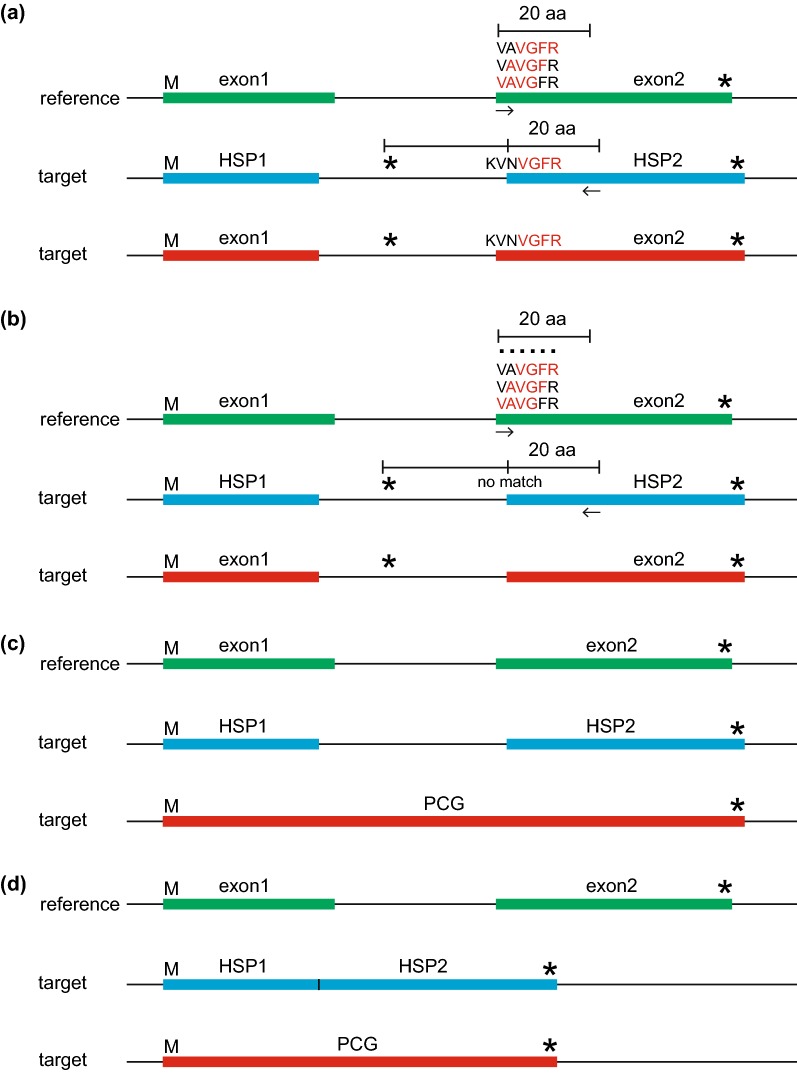
A description of the Intron Boundary Detection Algorithm (IBDA), which locates intron boundaries and detects intron loss for Protein-Coding Genes (PCGs). Panels (**a**) through (**d**) correspond to the description of this algorithm in the “Boundary detection algorithm” section of the main text. The blue bar denotes the original HSP resulting from TBLASTN search of the reference CDS. The red bar denotes the annotated PCG resulting from this algorithm. The green bar denotes the reference CDS with the highest percent identity to the target plastome. The “M” (methionine) and “*” denote the start (ATG) and stop (TAA/TAG/TGA) codons, as annotated by GBDA

Here we present PGA (Plastid Genome Annotator), a command line tool designed to conduct rapid, accurate, and flexible batch annotation of newly generated plastomes. A new approach, which we call reverse query-subject BLAST search, is used to locate genes, followed by algorithms that identify feature boundaries [including for genes, introns, and the Inverted Repeat (IR)] as well as intron loss (Figs. 1, 2 and 3). In reverse query-subject BLAST, the annotated reference plastome(s) is/are used as the query sequence and the unannotated target plastome(s) is/are used as the subject sequence to locate genes in the target plastome(s). Below we demonstrate the speed and utility of PGA through detailed comparisons with other existing tools (Table 1).

**Table 1:** Comparison of existing plastome annotation tools

Tools	Operating system	User interface	Time	Approach	Post-annotation algorithms for identification of feature boundaries	Output file format	Log file	References
DOGMA	Windows, Linux, Mac	Web	5–10 min	Target against reference	No	table	No	Wyman et al. [12]
CpGAVAS	Windows, Linux, Mac	Web	~ 1 h	Target against reference	No	GFF3 and GenBank	No	Liu et al. [13]
Plan	Windows, Linux, Mac	Console	~ 30 s	Target against reference	No	tbl	Yes	Huang and Cronk [16]
Verdant	Windows, Linux, Mac	Web	10–30 min	Target against reference	Yes	GFF3	No	McKain et al. [14]
GeSeq	Windows, Linux, Mac	Web	6 s–13 min	Target against reference	No	GenBank	No	Tillich et al. [15]
PGA	Windows, Linux, Mac	Console	~ 20 s	Reference against target	Yes	GenBank	Yes	This study

For PGA, a laptop equipped with 2.5 GHz 4-core Intel core i3 processors and 8 GB memory was used. Runtimes for other tools were derived from the corresponding references. The phrase “target against reference” signifies a BLASTN or TBLASTN search of a target plastome against an annotated reference plastome, whereas “reference against target” signifies a BLASTN or TBLASTN search of an annotated reference plastome against a target plastome

## Implementation

PGA is open-source and written in Perl. The core of PGA includes the reverse query-subject BLAST search approach to locate genes and boundary detection algorithms to identify feature boundaries as well as intron loss.

### Locating genes

BLASTN searches of a reference nucleotide database are used to locate rRNA and tRNA genes in target plastomes (Fig. 1). For protein-coding genes (PCGs), BLASTN and TBLASTN searches are conducted (Fig. 1). Any PCGs with a TBLASTN percent identity greater than the changeable threshold value (default = 40%) are annotated in the target plastome. If more than one reference plastome is used, each rRNA, tRNA or PCG with the highest BLASTN/TBLASTN percent identity is used to initially identify its position as the high-scoring segment pair (HSP) in the target plastome.

The genes *rpl16*, *petB* and *petD* form a special case. Each of these genes possesses a short first exon (6–9 bp in length) and a much longer second exon. BLASTN and TBLASTN are able to easily locate the second exon, but the first exons are too short to be detected. Because these first exons are highly conserved (for example, each possesses the same sequence across angiosperms with rare exceptions), a search of the region upstream of exon 2 in each gene is performed, using the exon 1 sequence of the reference plastome(s) as a probe.

### Boundary detection algorithms

To annotate feature boundaries correctly, three algorithms are applied to (1) determine start and stop codons, (2) locate intron–exon boundaries and detect intron loss, and (3) identify the boundaries of the inverted repeat (IR) (Fig. 1). The coordinates of HSPs acquired from TBLASTN search are used as preliminary data. PGA then uses the Gene Boundary Detection Algorithm (GBDA, Fig. 2) to identify start codon and stop codon for PCGs. To detect the stop codon, the GBDA search begins from the 5′ end of the HSP, and the first identified stop codon is returned as the annotated stop codon (Fig. 2). The proper start codon is identified via searching near the 5′ end of the HSP: (a) if the first amino acid of the HSP is methionine, its corresponding “ATG” will be annotated as the start codon (Fig. 2a); (b) if the first amino acid of the HSP is not methionine, PGA will search for methionines in the region between the first detected in-frame stop codon upstream of the HSP and the 20th amino acid of the HSP, and the one that is closest to the stop codon will be annotated as the start codon (Fig. 2b); (c) if no suitable methionine is detected in (a) or (b), PGA will use the first four amino acids (“VAVG”) of the reference CDS as a probe to search across the same region defined in step (b), with a search from right to left (Fig. 2c). If this fails to find a match, the four amino acid window is moved downstream in the reference CDS by a step of one amino acid (the probe is changed into “AVGF”), and so on to the 20th amino acid of the reference CDS. If this strategy yields an appropriate match (with the probe of “VGFR” after the four amino acid window being moved downstream by two steps in the reference plastome; Fig. 2c), PGA will treat the position of the first matched amino acid of the probe in the target plastome as the starting point (the “V” of “VGFR” in the target plastome; Fig. 2c). The position of the amino acid that corresponds to the “start codon” (the first “V” of “VNVGFR” in the target plastome) is then identified by moving left from this starting point by the number of steps (two steps) that the four amino acid window was shifted in the reference CDS. PGA then annotates the codon at this position as the “start codon”. If multiple putative start codons are detected, the one that is closest to the upstream stop codon will be annotated. (d) If strategy (c) fails to identify a start codon, the first amino acid of the HSP will be tentatively annotated as the putative “start codon” (Fig. 2d). If the “start codon” is identified via strategies (c) or (d), the annotated PCG for the gene in question will be noted in the log file to allow for manual verification. A similar algorithm to GBDA is applied to identify the annotation boundaries for rRNA genes and for tRNA genes without introns (Fig. 2c, d); this algorithm uses the first 9 nt from the first 30 nt at both ends of the reference RNA gene as probes. The search region is restricted to the first 30 nt at both ends of the HSP plus the adjacent 30 nt in the upstream and downstream regions.

PGA also uses the Intron Boundary Detection Algorithm (IBDA) to locate intron–exon boundaries and detect intron loss (Fig. 3). In IBDA, PGA (a) first identifies the existence of an intron due to the fact that a stop codon(s) is/are found in the region between the 3′ end of HSP1 and the 5′ end of HSP2 in the same reading frame as HSP1 and HSP2, or because the length of this region is not a multiple of three (Fig. 3a). PGA then uses the first four amino acids (“VAVG”; Fig. 3a) of exon2 of the reference CDS as a probe to search the region between the first detected stop codon upstream of HSP2 and the 20th amino acid of HSP2, with the search from right to left. If this fails to find a match, the four amino acid probe window is moved downstream by one amino acid in exon2 of the reference CDS (the probe is changed into “AVGF”; Fig. 3a), and so on until the 20th amino acid of the exon2 of the reference CDS. If this succeeds in locating a match (with the probe of “VGFR” after the four amino acid window being moved downstream by two steps in the reference plastome; Fig. 3a), PGA will treat the position of the first matched amino acid of the probe in the target plastome as the starting point (the “V” of “VGFR” in the target plastome). The position of the first amino acid of exon2 (the first “V” of “VNVGFR” in the target plastome) is then identified by moving left from this starting point by the number of steps (two steps) that the four amino acid window was shifted in the reference CDS, then the position of the first codon of exon2 is identified. The intron/exon2 boundary is finally identified through moving left from the position of the first codon of exon2 by the number of split-codon nucleotides, which is determined by dividing the length of exon2 in the reference CDS by three. The exon1/intron boundary is identified using the same process. (b) If strategy (a) fails to identify the intron boundaries, the first amino acid of HSP2 is tentatively annotated as the first codon, and the intron/exon2 boundary is identified through moving left from the position of the first codon by the number of split-codon nucleotides (Fig. 3b). This annotated PCG is then added to the log file for manual verification. Intron loss is detected in one of two ways: (c) if no in-frame stop codon exists between the 3′ end of HSP1 and the 5′ end of HSP2 (Fig. 3c), or (d) if the location of the 3′ end of HSP1 is the same as that of the 5′ end of HSP2, an intron loss event is noted in the log file and the joined exons are annotated as a single CDS (Fig. 3d). A similar algorithm to IBDA is applied to identify intron–exon boundaries for intron-containing tRNAs (Fig. 3a, b); it differs by using the first 9 nt from the first 30 nt at both ends of each reference tRNA exon as probes. The search region is restricted to the first 30 nt at both ends of the HSP1 and HSP2 plus their adjacent 30 nt in the upstream and downstream regions. IR boundary annotation is accomplished via a self-BLASTN search. One parameter can be adjusted to determine the IR boundaries: minimum allowed IR length (default = 1000).

### Detecting pseudogenes

To detect putative pseudogenes, PGA uses a parameter ([-q -qcoverage], optional: [default: 0.5,2]). Briefly, this parameter is determined by dividing the length of the annotated gene by that of the reference gene. The annotated genes with a query coverage less or greater than each of the two changeable threshold values will be added to the warning log file. Because pseudogenes can be highly variable among plastomes, users can adjust these two threshold values to satisfy their own needs. It is important to note that a pseudogene may fail to be identified using poorly fitting threshold values.

## Results and discussion

### Example

**Fig. 4:**
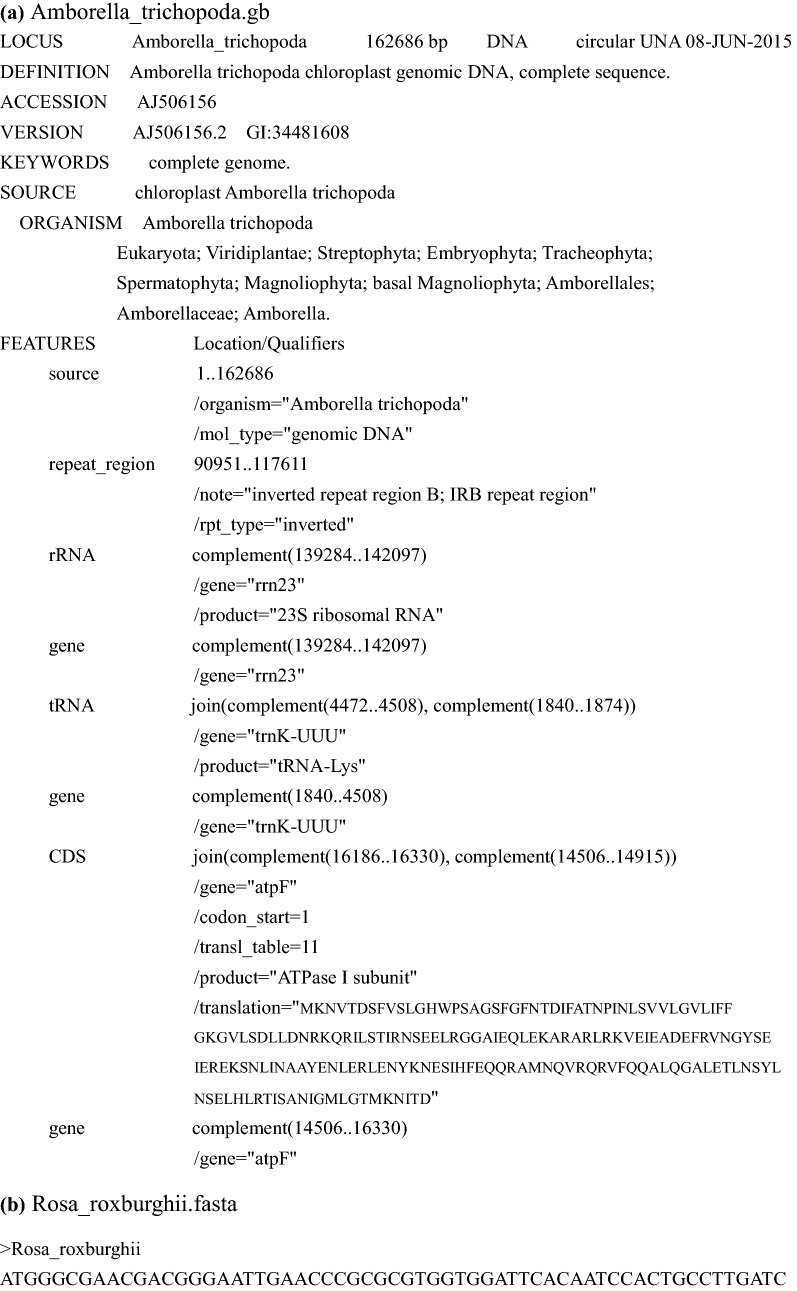

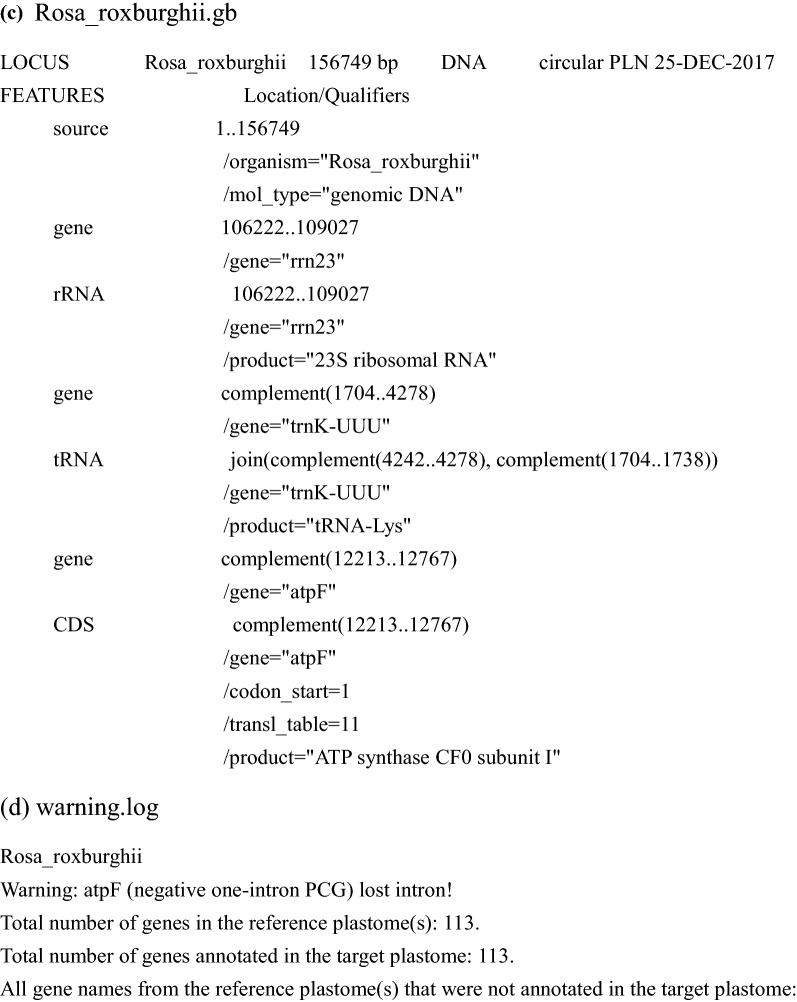
Annotation of the *Rosa roxburghii* plastome using PGA. **a** “Amborella_trichopoda.gb” shows the partial GenBank-formatted reference plastome of *Amborella trichopoda*, as revised from AJ506156. **b** “Rosa_roxburghii.fasta” shows the partial FASTA-formatted target plastome of *Rosa roxburghii*, revised from NC_032038. **c** “Rosa_roxburghii.gb” shows the output GenBank-formatted file containing partial annotation information for the target plastome of *Rosa roxburghii*. **d** “warning.log” shows warning and statistical items during the annotation of the target plastome of *Rosa roxburghii*. The log file indicates the loss of the *atpF* intron in *Rosa roxburghii*. There are 113 total genes in the reference and target plastomes

PGA consists of six steps (Fig. 1). The first two steps require user input, whereas the last four are automatic. As an example, we used PGA to annotate the target plastome of *Rosa roxburghii* Tratt. with the plastome of *Amborella trichopoda* Baill. as a reference (Fig. 4). Geneious R9 [17] was used to check flagged annotations.

*Step* 1 Preparation of GenBank-formatted reference plastomes

It is possible to use available GenBank-formatted reference plastomes within PGA, but we encourage users to prepare reference plastomes from relatives of the target taxa (Fig. 1). Reference plastomes, whether acquired from GenBank or other sources, must be carefully checked, especially to ensure that the indispensable “/gene” qualifier is present for each gene (Fig. 4a).

*Step* 2 Preparation of FASTA-formatted target plastomes

The target plastomes should be prepared in FASTA format, one sequence per file (Figs. 1 and 4b).

*Step* 3 Reference database generation

PGA uses annotation features (i.e. “gene”, “rRNA”, “tRNA” and “CDS”) from GenBank-formatted reference plastomes to generate a reference database with four components: RNA nucleotides, PCG nucleotides, coding sequence (CDS) amino acids without introns, and CDS amino acids with introns (Fig. 1). PGA parses reference plastomes based on “gene”, “rRNA”, “tRNA” and “CDS” qualifiers and extracts these features and their corresponding nucleotide sequences based on their coordinates. Then, nucleotide sequences of CDS are translated into amino acid sequences.

*Step* 4 BLAST search

Reverse query-subject BLAST searches are applied to locate genes in the target plastome (Fig. 1). Searching for a fixed number of genes takes full advantage of the conserved gene content of plastomes. BLASTN and TBLASTN [18] are used for searches of nucleotide and amino acid sequences, respectively. During searching, any PCGs with a TBLASTN percent identity less than the changeable threshold value (default = 40%) will be listed in the log file and will not be annotated.

*Step* 5 Determining feature boundaries

Gene and intron boundaries are initially determined from the BLAST search, and are then refined using the Gene Boundary Detection Algorithm (GBDA), which searches for start and stop codons (including those with non-ATG start codons), and the Intron Boundary Detection Algorithm (IBDA), which locates intron–exon boundaries and detects intron loss (Fig. 1). IR boundary annotation is accomplished via a self-BLASTN search. Details are provided in the section above (“Boundary detection algorithms”) and in Figs. 2 and 3.

*Step* 6 Generating GenBank and log files

The final step for each run is the generation of GenBank-formatted files and the log file (Figs. 1, 4c, d). To allow for manual verification, the log file will contain warnings concerning any unusual feature, including PCGs with non-ATG start codons and PCGs with a query coverage less or greater than each of the two changeable threshold values (default: 0.5, 2). For each target plastome, the log file also includes a list of the total number of genes in the reference plastome(s), the total number of genes annotated, and all gene names from the reference(s) that were not annotated in the target, to assist users in verifying questionable annotations.

### Overall performance

In order to measure the performance of PGA relative to other published tools, we re-annotated 20 gymnosperm plastomes and 20 angiosperm plastomes from GenBank using the properly annotated *Zamia furfuracea* Aiton plastome and *Amborella trichopoda* plastome as respective references (Tables 2, 3). We only conducted comparisons between PGA and GeSeq, because the performance of the most recently published GeSeq is equal or superior to other published tools [15]. In addition, PGA and GeSeq represent useful comparisons due to the full customizability of reference sequences by the user in both programs. In order to facilitate comparisons, PGA was run with default settings, and GeSeq was run in quick annotation mode. For rRNAs, tRNAs, and PCGs without introns, we compared the number of the missing annotated genes (MGs), wrongly annotated genes (WGs), wrongly annotated gene boundaries (WGBs) and correctly annotated genes (CGs). For tRNAs and PCGs with introns, we compared the number of missing annotated exons (MEs), wrongly annotated exons (WEs), wrongly annotated exon boundaries (WEBs) and correctly annotated exons (CEs).

**Table 2:** List of 20 gymnosperm plastomes from GenBank used to test the performance of PGA

Species	Family	Size (bp)	Accession no.
*Amentotaxus formosana*	Taxaceae	136,430	NC_024945
*Araucaria heterophylla*	Araucariaceae	146,723	NC_026450
*Callitris rhomboidea*	Cupressaceae	121,117	NC_034940
*Cephalotaxus wilsoniana*	Cephalotaxaceae	136,196	NC_016063
*Cryptomeria japonica*	Cupressaceae	131,810	NC_010548
*Cunninghamia lanceolata*	Cupressaceae	135,334	NC_021437
*Cycas taitungensis*	Cycadaceae	163,403	NC_009618
*Dacrycarpus imbricatus*	Podocarpaceae	133,811	NC_034942
*Dioon spinulosum*	Zamiaceae	161,815	NC_027512
*Ginkgo biloba*	Ginkgoaceae	156,988	NC_016986
*Juniperus communis*	Cupressaceae	128,334	NC_035068
*Metasequoia glyptostroboides*	Cupressaceae	131,887	NC_027423
*Podocarpus totara*	Podocarpaceae	133,259	NC_020361
*Retrophyllum piresii*	Podocarpaceae	133,291	NC_024827
*Sciadopitys verticillata*	Sciadopityaceae	138,284	NC_029734
*Sequoia sempervirens*	Cupressaceae	133,929	NC_030372
*Taiwania flousiana*	Cupressaceae	131,413	NC_021441
*Taxodium distichum*	Cupressaceae	131,954	NC_034941
*Torreya grandis*	Taxaceae	136,949	NC_034806
*Wollemia nobilis*	Araucariaceae	145,630	NC_027235

**Table 3:** List of 20 angiosperm plastomes from GenBank used to test the performance of PGA

Species	Family	Size (bp)	Accession no.
*Acorus gramineus*	Acoraceae	152,849	NC_026299
*Amborella trichopoda*	Amborellaceae	162,686	NC_005086
*Aralia undulata*	Araliaceae	156,333	NC_022810
*Buxus microphylla*	Buxaceae	159,010	NC_009599
*Calycanthus floridus*	Calycanthaceae	153,337	NC_004993
*Carludovica palmata*	Cyclanthaceae	158,545	NC_026786
*Chloranthus japonicus*	Chloranthaceae	158,640	NC_026565
*Ceratophyllum demersum*	Ceratophyllaceae	156,252	NC_009962
*Drimys granadensis*	Winteraceae	160,604	NC_008456
*Eucommia ulmoides*	Eucommiaceae	163,341	KU204775
*Hanguana malayana*	Hanguanaceae	163,231	NC_029962
*Larrea tridentata*	Zygophyllaceae	136,194	NC_028023
*Liquidambar formosana*	Altingiaceae	160,410	NC_023092
*Lupinus albus*	Fabaceae	154,140	NC_026681
*Nelumbo lutea*	Nelumbonaceae	163,206	NC_015605
*Potamogeton perfoliatus*	Potamogetonaceae	156,226	NC_029814
*Sapindus mukorossi*	Sapindaceae	160,481	NC_025554
*Trochodendron aralioides*	Trochodendraceae	165,945	NC_021426
*Typha latifolia*	Typhaceae	161,572	NC_013823
*Zingiber spectabile*	Zingiberaceae	155,890	NC_020363

**Fig. 5:**
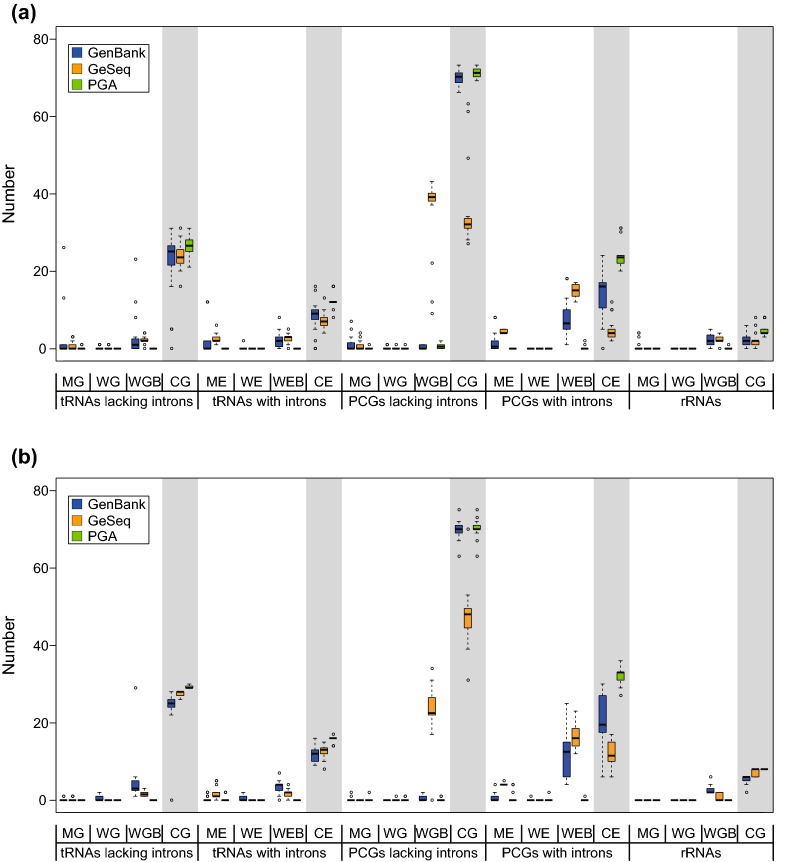
Boxplots comparing the performance of PGA and GeSeq. **a** Performance of PGA relative to GeSeq using the properly annotated *Zamia furfuracea* plastome as a reference. **b** Performance of PGA relative to GeSeq using the properly annotated *Amborella trichopoda* plastome as a reference. Thick lines within boxes are medians, the top and bottom of each box are quartile lines, and circles depict outliers. MG = missing annotated gene, WG = wrongly annotated gene, WGB = wrongly annotated gene boundary, CG = correctly annotated gene. ME = missing annotated exon, WE = wrongly annotated exon, WEB = wrongly annotated exon boundary, CE = correctly annotated exon

In general, PGA performed better than GeSeq (Fig. 5). For gymnosperms (Fig. 5a; Table 4), PGA and GeSeq produced similar average numbers of WGs/WEs for tRNAs lacking introns, tRNAs with introns, PCGs lacking introns, PCGs with introns and rRNAs, and similar average numbers of MGs for rRNAs. However, PGA annotated significantly lower average numbers of MGs/MEs than GeSeq for tRNAs lacking introns, tRNAs with introns, PCGs lacking introns and PCGs with introns, and lower average numbers of WGBs/WEBs than GeSeq for tRNAs lacking introns, tRNAs with introns, PCGs lacking introns, PCGs with introns and rRNAs. Importantly, PGA annotated higher average numbers of CGs/CEs than GeSeq for tRNAs lacking introns (26.60 vs. 23.70), tRNAs with introns (12.30 vs. 7.15), PCGs lacking introns (70.90 vs. 35.25), PCGs with introns (23.65 vs. 4.75) and rRNAs (4.70 vs. 2.20). For angiosperms (Fig. 5b; Table 4), PGA and GeSeq produced similar average numbers of WGs/WEs for tRNAs lacking introns, tRNAs with introns, PCGs lacking introns, PCGs with introns and rRNAs, and similar average numbers of MGs for tRNAs lacking introns, PCGs lacking introns and rRNAs. However, PGA annotated significantly lower average numbers of MEs than GeSeq for tRNAs with introns and PCGs with introns, and lower average numbers of WGBs/WEBs than GeSeq for tRNAs lacking introns, tRNAs with introns, PCGs lacking introns, PCGs with introns and rRNAs. Importantly, PGA annotated higher average numbers of CGs/CEs than GeSeq for tRNAs lacking introns (29.25 vs. 27.40), tRNAs with introns (15.85 vs. 12.50), PCGs lacking introns (70.15 vs. 47.35), PCGs with introns (32.15 vs. 12.05) and rRNAs (8.00 vs. 7.10). Furthermore, PGA had a lower interquartile range than GeSeq, indicating a higher percentage of consistently correct annotations (Fig. 5).

**Table 4:** Comparative performance of PGA and GeSeq in annotating plastomes

		Gymnosperms			Angiosperms		
		GenBank	GeSeq	PGA	GenBank	GeSeq	PGA
tRNAs lacking introns	MG	2.20	0.75	0.10	0.10	0.15	0.00
	WG	0.15	0.10	0.00	0.50	0.00	0.00
	WGB	2.85	2.2	0.00	4.65	1.70	0.00
	CG	21.60	23.70	26.60	24.00	27.40	29.25
tRNAs with introns	ME	1.65	2.60	0.00	0.30	1.75	0.20
	WE	0.10	0.00	0.00	0.50	0.00	0.00
	WEB	2.20	2.55	0.00	3.30	1.80	0.00
	CE	8.35	7.15	12.30	12.00	12.50	15.85
PCGs lacking introns	MG	1.15	0.65	0.05	0.15	0.00	0.10
	WG	0.10	0.05	0.05	0.00	0.05	0.10
	WGB	0.40	35.65	0.55	0.45	23.00	0.10
	CG	69.95	35.25	70.90	69.75	47.35	70.15
PCGs with introns	ME	1.55	4.35	0.00	0.70	4.05	0.50
	WE	0.00	0.00	0.00	0.05	0.00	0.10
	WEB	7.90	14.70	0.15	11.60	16.55	0.05
	CE	14.35	4.75	23.65	20.40	12.05	32.15
rRNAs	MG	0.60	0.00	0.00	0.00	0.00	0.00
	WG	0.00	0.00	0.00	0.00	0.00	0.00
	WGB	2.25	2.55	0.05	2.60	0.90	0.00
	CG	1.90	2.20	4.70	5.40	7.10	8.00

Numbers represent mean values

MG = missing annotated gene, WG = wrongly annotated gene, WGB = wrongly annotated gene boundary, CG = correctly annotated gene. ME = missing annotated exon, WE = wrongly annotated exon, WEB = wrongly annotated exon boundary, CE = correctly annotated exon

### Recommendations for using PGA

1. Users should carefully check the GenBank-formatted reference plastome. PGA is packaged with several properly annotated plastomes, and it is thus possible for users to use PGA to re-annotate a plastome that is intended to be used as a reference, in order to correct possible inaccuracies.
2. It is important that users select a reference plastome that contains sufficient numbers of annotated genes for the target taxa. The number of genes in the reference plastome(s) should equal or exceed the number in the target plastome(s). If the number of genes in the target is uncertain, it may be best to use multiple reference plastomes. The *Amborella trichopoda* (AJ506156) and *Zamia furfuracea* (JX416857) plastomes included within PGA are examples of plastomes that contain the highest gene numbers among known angiosperms and gymnosperms, and as such it is recommended that they be included as references during PGA runs.
3. We do not recommend annotating highly incomplete plastomes using a complete reference plastome, because BLAST may annotate some genes redundantly (i.e., BLAST may return hits for genes that were not sequenced or are otherwise absent in the incomplete plastome, resulting in spurious annotations). To annotate highly incomplete plastomes or plastome segments, we recommend using progressiveMauve as implemented in Mauve 2.4.0 [19] to align the incomplete plastome to the reference plastome, followed by the use of the corresponding homologous block of the reference plastome as the reference for annotation in PGA.
4. We suggest that users carefully check highly divergent or otherwise unusual target plastomes for incorrect annotations. This is particularly important for plastomes with a high degree of gene loss, pseudogenization or sequence divergence.

## Conclusions

Comparisons with other plastome annotation tools demonstrate the speed and high annotation accuracy of PGA. Importantly, PGA is also highly flexible, as demonstrated by the annotation of the *Rosa roxburghii* plastome using the phylogenetically distant *Amborella trichopoda* plastome as a reference. For projects in which multiple plastomes are generated, the time savings for high-quality plastome annotation are especially significant.

## Availability and requirements

Project name: PGA-Plastid Genome Annotator

Project home page: https://github.com/quxiaojian/PGA

Operating systems(s): Platform independent

Programming language: Perl

Other requirements: Perl 5, BLAST 2.5.0 or higher

License: GPL-3 (https://www.gnu.org/licenses/gpl-3.0.en.html)

Any restrictions to use by non-academics: none.

## Data Availability

The program, user manual and example data sets are freely available on https://github.com/quxiaojian/PGA.

## Abbreviations

PGA: Plastid Genome Annotator
IR: inverted repeat
PCGs: protein-coding genes
HSP: high-scoring segment pair
GBDA: Gene Boundary Detection Algorithm
IDBA: Intron Boundary Detection Algorithm
CDS: coding sequence MGs: missing annotated genes
WGs: wrongly annotated genes
WGBs: wrongly annotated gene boundaries
CGs: correctly annotated genes
MEs: missing annotated exons
WEs: wrongly annotated exons
WEBs: wrongly annotated exon boundaries
CEs: correctly annotated exons
